# Theory of Mind and Young Children’s Behaviour: Aggressive, Victimised, Prosocial, and Solitary

**DOI:** 10.3390/ijerph20105892

**Published:** 2023-05-20

**Authors:** Katie Rix, Claire P. Monks, Sarah O’Toole

**Affiliations:** 1 School of Psychology & Counselling, Faculty of Arts & Social Sciences, The Open University, Milton Keynes MK7 6AA, UK; 2 Institute for Lifecourse Development, School of Human Sciences, University of Greenwich, London SE10 9LS, UK; 3 Department of Civil, Environmental & Geomatic Engineering, Faculty of Engineering Science, University College London, London WC1E 6BT, UK

**Keywords:** Theory of Mind, young children, behaviour, aggressive, prosocial, solitary, victimisation

## Abstract

Theory of mind (ToM) undergoes significant developments during childhood, particularly between the ages of four and seven years. A growing body of research has indicated that children’s social understanding may be related to their social behaviour with peers, in line with Theory Theory which proposes that children’s social cognition is influenced by and influences their peer interactions. The current study examined the relationship between ToM and behaviour among 193 children aged 4–7 years. Children carried out a battery of ToM tasks, and teaching staff reported on children’s aggressive, prosocial, and solitary behaviour and victimisation experiences. Aggression was not directly related to ToM; prosocial behaviour was positively associated with ToM for girls but not boys. Solitary behaviour and victimisation were negatively related to ToM. When this was broken down by gender, there was only a significant association between solitary behaviour and ToM for boys. When controlling for the relationship between behaviours, the only significant predictor of ToM was solitary behaviour for boys. ToM was also a significant predictor of solitary behaviour for boys, demonstrating that there is a bidirectional relationship at play. The findings highlight the importance of looking across these four behaviour types and understanding the relationship between behaviour profiles and ToM for boys and girls separately.

## 1. Introduction

The development of Theory of Mind (ToM), the understanding that others have their own thoughts, beliefs, and desires that may differ from one’s own [1], has been extensively researched. Between the ages of four and seven years, children show significant developments in ToM, although there is individual variation [2]. These advances have important consequences for children’s social behavioural development [3]. According to models of children’s social behaviour development, however, ToM does not just predict children’s social behaviour, but children’s early social experiences influence their ToM development [3,4]. That is, the relation is bidirectional. The majority of research has so far focused on specific categories of peer experiences, such as aggressive, victimised, prosocial, and solitary behaviours, rather than considering associations between these and how these various experiences compare with each other. Therefore, the extent to which these joint experiences and behaviours relate to ToM warrants further exploration.

### 1.1. Changing Social Experiences

Starting compulsory schooling is a considerable social milestone for children, one where they encounter numerous social changes, particularly in their peer interactions [5]. Research has shown that these peer relationships during the early school years play an important role in children’s social skills development [6]. 

### 1.2. The Development of Theory of Mind

Alongside these social changes, young children are also developing cognitively. One area of significant focus among researchers has been Theory of Mind (ToM), which refers to the ‘ability to represent others’ mental states’ [7], p. 2. One way of assessing ToM ability among children has been the use of false belief tasks. These tasks typically employ fictional scenarios to see whether a child can infer that a character who is holding a false belief will act according to their own false belief rather than a fact (which is known to the child) [8]. Resources such as pictures, videos, and dolls are used to see how far children can predict the knowledge, behaviour, and emotion of others. By age four to five, research has shown that typically developing children demonstrate understanding that an actor will behave in accordance with their false belief [1]. Whilst some researchers have suggested that even younger children are able to demonstrate understanding of false beliefs, others have suggested that the competence shown by infants has not yet developed into an understanding of false belief [9]. Further development of ToM is measured through second-order false belief tasks (e.g., I think that X thinks that Y thinks), which is considered as demonstrating a more complex understanding of ToM [10]. Sullivan et al. [11] explored second-order false belief tasks in young children and found that approximately half of the pre-schoolers (aged four to five years) and all of the kindergarteners (aged five to six years) could demonstrate this level of understanding. Other researchers have explored children’s understanding of double-bluff as a means of examining more complex aspects of ToM [12] and have demonstrated increasing ToM abilities with age across these tasks [12]. Hughes et al. [13] highlight the importance of aggregating across multiple individual measures of ToM to ensure improved reliability to understand children’s conceptions of mind. 

### 1.3. Theory of Mind and Changing Social Experience

Research has suggested that ToM is important for multiple purposes such as the development of social skills [14], social competence [15] and peer acceptance [16]. As explained by Lane and Bowman [17], this positive association between ToM and aspects of a child’s social skills may occur as a result of a bidirectional relationship between the child and their social environment. Several theories have been proposed to explain the relationship between social experience and children’s ToM development. A prominent account is Theory Theory [18,19], which argues that children’s conceptual ToM development undergoes refining and reformulation, similar to that of scientific theories. According to Theory Theory, social experiences are central to children’s shift in conceptual understanding, as social experiences provide children with information that cannot be accounted for by their present ToM, and this will require them to revise and improve that theory. This shows that the richness of the social environment is important for the development of ToM but also that ToM is important for the way in which children engage with their social environment. There is a body of research that has examined the association between ToM and children’s social behaviour, indicating that there are important links between social understanding and social interactions.

### 1.4. Conceptualisations

#### 1.4.1. Aggression and Victimisation

One form of behaviour shown between peers is aggression. As explained by Underwood et al. [20], although definitions of aggression vary, they tend to share two main features: that the behaviour is intended to cause harm and that it is considered hurtful by the recipient. In addition to physical aggression, multiple distinctions have been used in the study of aggression, such as indirect aggression (involvement of a third party), relational aggression (damage to peer relationships), and social aggression (damage to self-esteem or social status) [21,22]. Despite these various distinctions, researchers have shown considerable overlap between the various forms of aggression and, for this reason, have often collapsed the various forms of aggression into a single behaviour [23,24,25]. Furthermore, research has shown a consistent negative relationship between aggression and ToM. In particular, Wang et al.’s [26] recent meta-analysis found that, with the exception of bullying, ToM was negatively associated with all forms of aggression. Similarly, Baker et al. [27] showed the moderating role of ToM in both physical and relational aggression when comparing children from different socio-economic backgrounds, and Olson et al. [28] reported a negative association between aggression and ToM. Applying Theory Theory, the negative association between ToM and aggression may occur because children who have poorer ToM may have less rewarding peer relationships (which may lead to them behaving aggressively), and by behaving aggressively, children have less rewarding peer interactions, which will lead to poorer ToM development. It is worth noting that, although aggression appears to be negatively associated with ToM, there is some research that has found that this is not the case among all age groups for all forms of aggression—for example, bullying (as a subset of aggression, characterised by repetition and the exploitation of an imbalance of power by the perpetrator) has been found to be positively associated with ToM during middle childhood [29].

When considering victimisation, Monks et al. [30] found that there was no association between ToM and victimisation, among four- to six-year-olds. This may be because victimisation tends to be relatively short-lived for many children during early childhood [30,31,32,33], and therefore, these interactions do not prevent the child from encountering other positive social experiences, allowing them to develop their ToM. 

#### 1.4.2. Prosocial Behaviour 

Prosocial behaviour typically includes a range of behaviours such as sharing, cooperating, helping, and comforting peers, regardless of motives [34]. Some researchers have also considered the importance of relational inclusion as a prosocial behaviour, where a child invites another child to join a group or game [35,36]. As demonstrated by Wardle et al. [37], there are high levels of consistency across subtypes of prosocial behaviour, and therefore, this suggests that these different forms of prosocial behaviour tap into the same underlying concept.

There is a growing body of literature that has examined the relation between ToM and prosocial behaviour. Studies have indicated that higher levels of prosocial behaviour are related to superior levels of ToM performance among four- to six-year-olds [30,38]. The positive association between ToM and prosocial behaviour may be explained by Theory Theory, whereby children’s ToM may develop as they encounter more social situations (and vice versa; their ToM may influence their peer behaviour). However, there are contradictory findings regarding gender differences: Yagmurlu [39] found a positive relation between ToM and prosocial behaviour only for boys, whereas Walker [40] found that ToM positively predicted prosocial behaviour among three- to five-year-old girls and not boys. Therefore, it appears that gender may play a moderating role in this development. This can be explained by the idea that social norms can impact children’s behaviour [41] which may then in turn influence their ToM development. However, Imuta et al. [42] found that the link between prosocial behaviour and ToM occurred for both males and females but was stronger in children aged six and over. As such, there is a scope to explore the role of gender in prosocial behaviour and ToM further.

#### 1.4.3. Solitary Behaviour

Solitary behaviour in young children occurs when they stay on their own, despite the presence of peers [43]. Many terms are used to describe solitary behaviour, many of which are based on observed or assumed reasons/functions for children’s solitary behaviour. For instance, ‘active isolation’ [44] or ‘peer rejection’ [45] refers to a child being isolated by peers. In contrast, others have identified ‘social withdrawal’, which is where a child makes a choice to stay by themselves. Whether this is because they prefer solitary activities and are unsociable [46] or are socially disinterested [47], their behaviour still appears as solitary. Alternatively, a child may want to interact with others but may show ‘shyness’ [47] or ‘anxious-solitude’ [48], meaning their desire for social interaction is compromised by social fear and wariness. There have also been further developments of terms to include ‘avoidance’, where children seek out solitary situations [49]. 

Some researchers have indicated that the motivation for solitary behaviour can affect the relationship with ToM [15,50,51]. In contrast, others have suggested that solitary behaviour and the act of being alone, regardless of the reason for this, is negatively associated with ToM. For instance, in work by Walker [40], solitary behaviour (withdrawn or shy) was related to poorer ToM in three- to five-year-old boys (but not girls). In much of this research, the focus on attention or motivation removes the importance of the act of being alone and how this itself may be influenced by or influence the child’s developing ToM. As shown by Spangler and Gazelle [45], the associations between the different motivations for solitary behaviour are very strong, and they question whether unsociability should be distinguished as a separate construct from other solitary behaviours (shyness and exclusion). As highlighted by Coplan et al. [52], few researchers have shown benefits of spending time alone. As such, it is possible that being alone may compromise ToM development by limiting the number of direct social experiences a child has, regardless of whether they are observing or are motivated to interact. Zava et al. [53] found that, whilst children (aged 2–6 years) do have some distinctive understanding about subtypes of withdrawn behaviour by peers, there were no significant differences in peer reports of affiliative preferences, perceived social standing, or perceived negative impacts in hypothetical peers across shy, avoidant, and unsociable behaviours. This suggests that, at least during early childhood, peers behave in the same way towards children exhibiting solitary behaviour, regardless of its form. Thus, it may be the case that, during early childhood, children who behave in a solitary way may have similar peer experiences despite the underlying reason for this behaviour. In line with the reciprocal environment in Theory Theory, we may therefore expect that, if being alone decreases the richness of the child’s environment, there may be a negative association between ToM and solitary behaviour during early childhood. It is acknowledged that understanding how ToM relates to the different forms of solitary behaviour would be useful in further research, but for the purposes of the current study, it is proposed that solitary behaviour is measured as an overall construct, in line with aggressive, victimised, and prosocial behaviour. 

### 1.5. Looking across Behaviour Types

As discussed, models of social behaviour development have posited that ToM development is associated with a range of social behaviours and experiences, including prosocial, aggressive and solitary behaviour, and victimisation and that social-cognitive and behavioural factors interact [3,4,40]. Whilst research has often studied the association between these behaviours and ToM in isolation, more researchers have considered the relationship between various types of behaviour and ToM. 

One example of this is the complex relationship between aggressive and prosocial behaviour [54]. Children have been found to use both aggressive and prosocial strategies interchangeably and skillfully to gain dominance, control resources, influence play, and achieve a high status among peers [55,56,57,58]. Hawley [59] refers to these children as bistrategic controllers. Despite their tendency for high levels of relational and physical aggression, these children are often well liked by peers and experience higher levels of intimacy and fun in their friendships, although their friendships are also characterised by high levels of conflict [60]. Bistrategic controllers contradict the traditional view of aggressive children as having poorer social skills. Indeed, bistrategic controllers have well-developed social skills and moral understanding [61]. There is some evidence that the level of prosocial behaviour and aggression interact in the relationship with ToM. Renouf et al. [62] found that the relationship between ToM and aggression was moderated by the level of prosocial behaviour in children; for those low or moderate in terms of prosocial behaviour, there was a significant positive relation between ToM and indirect aggression, but this was not the case for those high in terms of prosocial behaviour. Therefore, it is possible that children who are low to average in terms of prosocial behaviour may employ their social understanding in the use of indirect aggression, whereas children who are prosocial are less likely to be indirectly aggressive, regardless of their level of ToM [62]. 

There is also emerging evidence indicating that there may be an interaction between aggression and victimisation in their relationship with ToM. Some children are aggressive to others and are also victims of aggression [63]. Based on research by Renouf et al. [64], it is possible that victimised aggressors may be more likely to show poorer ToM. They found that children who have poorer ToM and experience harsh treatment from peers (peer-victimisation) are more likely to display reactive aggression (aggression in response to a perceived provocation). In addition to this, studies have found that children classed as bully-victims (children who both bully others and are victims of bullying) during adolescence showed the poorest ToM performance at age five [65]. They argue that children who have poorer ToM may find it difficult to take others’ perspectives and may be viewed as behaving provocatively. They may also be more likely to be reactively aggressive, as they may interpret others’ behaviours as being aggressive based on their own (negative) peer experiences. Furthermore, children who are aggressive and victims of aggression may experience negative or limited peer interactions, which may influence their subsequent opportunities to develop an understanding of others’ minds. 

Aggressive behaviour has also been associated with some forms of solitary behaviour. For instance, children who use aggression in conflict situations (reactive aggression) have been found to experience greater peer rejection [66]. This may limit children’s interactions with their peers, reducing their opportunities to engage in imaginative play and interactions that highlight differences in peer’s minds, and thus influence ToM development [67]. Similarly, children who display both aggressive and withdrawn behaviour have been found to have more social skill problems [68] and more difficulties in peer and teacher relations than non-aggressive and non-withdrawn peers [69]. These social skills may relate to ToM and, in line with Theory Theory, mean that this remains less developed in children displaying these behaviours.

A range of other research has looked at the relationship between the various behaviour types. For instance, children who have a higher preference for being alone have been found to demonstrate lower levels of prosocial behaviour [70]. In conflict situations, including being teased by a peer or socially excluded, children aged four to six years engaged in both more aggressive (e.g., hitting) and prosocial (e.g., negotiation) strategies than three-year-old children [71]. Others such as Oh et al. [72] have looked across victimisation, social withdrawal, and prosocial behaviour to understand the trajectories of behaviour in older children. These various studies, and the knowledge that some children show complex behavioural profiles that vary across contexts and recipients [73], highlight the importance of looking across these in the study of ToM. 

### 1.6. Research Gaps and the Current Study

The review of research has highlighted two main points: First, there are associations between ToM and the separate constructs of aggression, victimisation, and prosocial and solitary behaviours in young children, and in some cases, this varies by gender. Second, studies have demonstrated the importance of looking across behaviours rather than looking at them separately to understand the child’s peer interactions and how these may relate to ToM in children aged four to seven years. The current study addresses these gaps, examining gender differences in the association between ToM and behaviour and how ToM is associated with varied behaviours in childhood.

## 2. Materials and Methods

### 2.1. Participants

The sample included 193 children (Girls *n* = 97) attending three mainstream primary schools in the Southeast of England, from lower to middle class areas. Children were four to five years old (from Reception classes; Male: 50; Female: 52: Total *n* = 102) and six to seven years old (from Year Two classes; Male: 46, Female: 45, Total *n* = 91), with a mean age of 71.98 months (*SD* = 12.39). The class teachers (*n* = 8, all female) and Teaching Assistants (*n* = 7, 6 were female) of those children participating were also recruited. Power analysis in SPSS showed that, even when split by gender, the sample sizes were sufficient to detect effects in partial correlations and Multiple Regression [74]. 

### 2.2. Measures

Verbal ability: Children’s verbal ability was assessed individually using the British Picture Vocabulary Scale (BPVS) [75]. Standardised scores were used for the purposes of analysis.

Theory of Mind: A battery of tasks was used. Scores on the ToM battery were summed, giving a potential range from 0 (none correct) to 25 (all correct). All measures of ToM were significantly moderated and correlated with the overall ToM battery (all at *p* < 0.001), demonstrating the importance of combining these measures to gain a holistic view of a child’s ToM.

Deceptive box task—The Smarties task [76] was employed to assess first-order false belief understanding. To ensure familiarity with the container, an egg box was used instead of a Smarties tube, because we found from piloting that many children did not recognise Smarties tubes (similar to Hughes [77] and Razza & Blair [78]. 

Unexpected transfer task—The Sally and Anne task [79] was used to assess first-order false belief understanding. 

Second-order false belief—The Sally and Anne through the window task (as described in Monks et al. [80]) assessed children’s understanding of second-order false beliefs.

Each task was filmed with a researcher demonstrating the tasks for the children and asking the questions in order to ensure the parity of delivery across settings. Children were shown these videos on a laptop. Research has shown that typically developing children do not show significantly different performances on ToM tasks that are presented in different formats (video/line-drawing/spoken) [81].

The Theory of Mind Test (TMT)—The TMT [12] was employed to examine different aspects of ToM. Following Caputi et al. [82], 22 of the 30 pictorial items were administered to each child. Two items from each of the following eight sections were administered in the following order: (i) Perspective taking Level 1; (ii) Perspective taking Level 2; (iii) Intention understanding; (iv) Ignorance understanding; (v) First-order false belief understanding; (vi) Appearance–reality distinction; (vii) Understanding lies; (viii) Understanding jokes. Three items from each of the following two sections were administered: (ix) Second-order false belief understanding; (x) Understanding double bluff. Children were also asked to explain their answers to the double bluff questions to ensure that they were demonstrating understanding of mind.

Behaviour: Children’s aggressive, prosocial, and solitary behaviours and experiences of victimisation were assessed using class teacher and Teaching Assistant (TA) reports. The participants completed a questionnaire which had four items for each behaviour (sixteen in total). They rated each child’s behaviour on a Likert scale from 1 (never/almost never true) to 5 (always/almost always true). 

Total scores for each behaviour category were created separately for teachers and TAs, and inter-rater reliability was calculated. Intraclass Correlations were significant for all categories of behaviour (*p* < 0.001), and, with the exception of solitary behaviour, 95% confidence intervals showed moderate reliability between the two adult informants. For solitary behaviour, there was poor to moderate agreement (*ICC* = 0.44). As such, the percentage agreement was explored for each individual solitary behaviour. These were all higher than 70% agreement (complete or one-point difference). Whilst these findings show that there is not complete agreement between teachers and TAs (which is a useful area of future analysis), this is likely to be due to the different scenarios they see children in and because of potential differences in these interactions [83]. Therefore, this highlights the importance of combining these different reports to gain a more accurate and holistic view of children’s behaviour. Correlations were also run with the teacher and TA total behaviour scores for each relevant subtype of behaviour (e.g., teacher reports of sharing, caring, helping, and group inclusion were each correlated with the total score of prosocial behaviour). All correlations were significant (*p* < 0.001) and moderate to high. This is with the exception of TA reports of helping (*r* = 0.303, *p* < 0.001) and active solitude (*r =* 0.495, *p* < 0.001). Whilst this highlights a potential area of future study, this also emphasises the importance of including these TA perspectives.

The total behaviour scores across teachers and TAs were averaged to provide one score per behaviour category (aggressive, victimised, prosocial, solitary behaviour) for each child. One class only received reports from their class teacher, so these were used. Possible scores ranged between 4 and 20 for each behaviour category. Reliability was further checked by including the eight reports (across teachers and TAs) within each behaviour type. All alphas ranged between 0.69 and 0.79. A reliability coefficient between 0.60 and 0.80 is considered moderate but acceptable [84]. As the summing of these variables within each behaviour category was to enable analysis across behaviour profiles, these were deemed acceptable. 

Items for *aggressive behaviour* were based on Monks et al. [80]: indirect relational (spreading rumours); direct relational (exclusion); physical (hitting, kicking, pushing) and verbal aggression (shouting at and calling names). (α = 0.76). Victimisation (being the victim of aggression) included complementary items addressing indirect relational, direct relational, and physical and verbal victimisation. (α = 0.77) [77]. Prosocial items were based on Crick et al. [85] and Greener [35]): sharing (is good at sharing and taking turns); caring (is kind to peers and cares for others when they are upset); helping (is helpful to peers); and including (approaches other children and asks if they would like to join in with them and their friends) (α = 0.69). Solitary behaviour was assessed using three items from Spangler and Gazelle [45]: shyness (when around peers, act shy, do not talk much, seem to be nervous or self-conscious, often play alone at break time, and may sit alone at lunch or not have anyone to talk to at lunch.); unsociable (play or stay alone, not because they are shy or afraid but because they like to play alone); active solitude (stay/play alone because they are left out when other children are talking and playing together). A fourth item was developed based on Rix et al. [86]: contextual solitude (stays/plays alone because the children they would like to play with are unavailable—e.g., absent or at a different lunch slot) (α = 0.73). 

### 2.3. Procedure

Ethical approval for the study was obtained from the University of Greenwich Ethics Committee (Reference 14.1.5.5). Consent was obtained from head-teachers and parents/guardians for child participation; assent was obtained from child participants. Child assessments were carried out individually in a quiet area of the school by trained researchers (*n* = 5, all female). Children were seen by the researchers twice during the assessment period for 20 min each time to ensure that sessions were not too long to maintain concentration. Assessments were administered in a set order. At the first assessment, children were administered the BPVS [75] and the TMT [12]. In the second assessment, children were administered the Sally and Anne task [79], the second-order False Belief task [30], and the Smarties task [76]. 

Teachers and TAs provided consent for their own participation and completed the questionnaires in their own time. 

## 3. Results

### 3.1. Behaviour Ratings

Adult ratings of children’s behaviour were highest for prosocial behaviour (*M =* 13.50, *SD* = 2.44), followed by aggressive behaviour (*M* = 8.01, *SD =* 2.96), victimisation (*M* = 7.34, *SD* = 2.43), and solitary behaviour (*M* = 7.30, *SD* = 2.51). 

*T*-tests revealed that girls received significantly higher prosocial scores than boys (*t* (191) *=* 3.961, *p* < 0.001 *r^2^* = 0.27 (girls: *M =* 14.19, *SD =* 2.47; boys: *M =* 12.80, *SD =* 2.21)). No other gender differences reached significance. 

### 3.2. ToM Performance

Children scored a mean of 15.64 out of 25 (*SD =* 4.71) on the ToM battery. ToM performance was significantly correlated with age (*r* = 0.562, *p* < 0.001) and BPVS score (*r =* 0.311, *n =* 193, *p* < 0.001). These variables were controlled for in subsequent analyses. A one-way ANCOVA controlling for age (measured in months) and BPVS score indicated that there was no gender difference in ToM performance (*F* (1, 189) = 1.50, *p* = 0.223, *η^2^* = 0.008 (boys: Mean = 15.14, *SD* = 4.92; girls: Mean = 16.14, *SD* = 4.47)). A one-way ANCOVA controlling for age and BPVS score indicated that there was a significant effect of age group on ToM performance (*F* (1, 189) = 19.46, *p* < 0.001, *η^2^* = 0.093 (4–5 year-olds: Mean = 12.96, *SD* = 4.06; 6–7 year-olds: Mean = 18.65, *SD* = 3.41)). 

### 3.3. Behaviour and ToM

Partial correlations were run to assess the relationship between children’s behaviour and ToM (controlling for age in months and BPVS score). There was a significant positive correlation between prosocial behaviour and ToM (*r* = 0.186, 189 df, *p =* 0.010). There were significant negative correlations between solitary behaviour and ToM (*r =* −0.249, 189 df, *p* < 0.001) and victimisation and ToM (*r =* −0.173, 189 df, *p =* 0.017). There was no significant relationship between aggressive behaviour and ToM (*r* = 0.088, 189 df, *p =* 0.225).

### 3.4. Relation between ToM and Behaviour by Gender

Partial correlations were conducted to examine the relationship between ToM performance and behaviour separately for boys and girls (see Table 1). For girls, there was a significant positive correlation between ToM and prosocial behaviour (*r* = 0.269, 93 df, *p* = 0.009). No other correlations reached significance. For boys, there was a significant negative correlation between solitary behaviour and ToM (*r =* −0.317, 92 df, *p* < 0.001). 

### 3.5. Hierarchical Multiple Regression

#### 3.5.1. A Model of Behaviour for Predicting ToM

A hierarchical linear regression was carried out with each behaviour type and ToM for the entire sample. Control variables (age in months and standardised BPVS score) were entered into the model first to account for potential confounding effects. Interaction effects between behaviours were also entered in a third block. Predictor variables were centered around the mean.

The full results of the hierarchical regression analyses are presented in Table 2. These show that the first block introducing age and standardised BPVS contributed significantly to the variance of the ToM (*F* (2, 190) = 66.407, *p* < 0.001), explaining 41.1% of the variance. Introducing the four behaviours into the model led to 45.8% of the variance being explained, and this increase was significant (Δ*R*^2^ = 0.047, *F* (4186) = 4.003, *p* = 0.004). Including the interactions in the model led to 47.6% of the variance being explained, but this increase was not significant (Δ*R*^2^ = 0.018, *F* (6180) = 1.011, *p* = 0.420).

When looking at the individual contributions of behaviours, prior to the introduction of the interactions (block 2), solitary behaviour negatively predicted ToM (*p* = 0.019). No other behaviours significantly contributed to the model. 

#### 3.5.2. A Model of Behaviour for Predicting ToM, by Gender

Two further hierarchical regressions were then conducted separately for each gender. The control variables of age and BPVS were entered in the first block, and the four behaviours were entered into the second block. Predictor variables were centered around the mean. Due to the insufficient sample size, it was not possible to include interactions of behaviour in the model when split by gender. 

Full results of the hierarchical regression analyses are presented in Table 3. These show that the first block introducing age and standardised BPVS contributed significantly to the variance in the ToM for both boys (*F* (2,93) = 33.245, *p* < 0.001) and girls (*F* (2,94) = 31.526, *p* < 0.001), explaining 41.7% and 40.1% of the variance, respectively. For boys, introducing the four behaviours into the model led to 49.0% of the variance being explained, and this increase was significant (Δ*R*^2^ = 0.073, *F* (4,89) = 3.179, *p* = 0.017). For girls, introducing the four behaviours into the model led to 45.0% of the variance being explained, but this increase was not significant (Δ*R*^2^ = 0.049, *F* (4,90) = 2.003, *p* = 0.101).

When looking at the individual contributions of behaviours, solitary behaviour negatively predicted ToM in boys only (*p* = 0.015). No other behaviours significantly contributed to the model. 

#### 3.5.3. Models using ToM to Predict Behaviours

To understand the relationship between behaviours and ToM further, hierarchical regressions were then run in the opposite direction with outcome variables of each behaviour type that had shown significant partial correlations (see Table 1). BPVS, age, and the other three behaviours were entered as predictors, with ToM being entered in the second block. All predictor variables were centered around the mean. Based on the partial correlation findings, this was split by gender for solitary and prosocial behaviour, but not victimisation. Splitting by gender reduced the sample size, and therefore, it was not possible to run interactions. 

##### Solitary Behaviour

Hierarchical multiple regressions were carried out separately for boys and girls to predict solitary behaviour. The age, standardised BPVS score, and behaviour scores (aggression, prosocial, and victimisation) were entered in the first block. The ToM score was entered in the second block. For boys, it was found that, jointly, the variables entered in the first block accounted for 27.7% of the variance in solitary behaviour (*R*^2^ = 0.277, *F* (5,90) = 6.907, *p* < 0.001) (see Table 4). There was a significant increase in the explained variance (27.7% to 32.4%) when ToM was added into the model for boys (Δ*R*^2^ = 0.047, *F* (1,89) = 6.161, *p* = 0.015). Each of the behaviours were significant predictors in solitary behaviour (aggressive behaviour: β = −0.526, prosocial behaviour: β *=* −0.265, victimisation: β = 0.482, all *p* < 0.01). ToM was also a significant predictor (β = 0.293, *p* < 0.05). 

A hierarchical multiple regression was conducted to predict solitary behaviour among girls. The age, standardised BPVS score, and behaviour scores (aggression, prosocial, and victimisation) were entered in the first block. The ToM score was entered in the second block. For girls, the variables in the first block accounted for 26.4% of the variance in solitary behaviour (*R*^2^ = 0.264, *F* (5,91) = 6.543, *p <* 0.001) (see Table 4). When ToM was entered into the model, the variance only increased from 26.4% to 27.2%, and this was not significant (Δ*R*^2^ = 0.007, *F* (1,90) = 0.882, *p* = 0.350). The only significant predictors were prosocial behaviour (β = −0.270, *p* < 0.05) and victimisation (β = 0.531, *p* < 0.001). 

##### Prosocial Behaviour

Hierarchical multiple regressions were carried out separately for boys and girls to predict prosocial behaviour. The age, standardised BPVS score, and behaviour scores (aggression, solitary, and victimisation) were entered in the first block. The ToM score was entered in the second block.

For boys, it was found that, jointly, the variables entered in the first block accounted for 33.9% of the variance in prosocial behaviour (*R*^2^ = 0.339, *F* (5,90) = 9.213, *p* < 0.001) (see Table 5). There was no significant increase in the variance explained when ToM was entered into the model (Δ*R*^2^ = 0.020, *F* (1,90) = 3.505, *p* = 0.064). Solitary behaviour (β = −0.210, *p* = 0.032), aggressive behaviour (β = −0.563, *p* < 0.001), and BPVS (β = 0.173, *p* = 0.028) were significant predictors of prosocial behaviour.

For prosocial behaviour in girls, the first block of the model explained 47.8% (*R*^2^ = 0.478, *F* (5,91) = 16.648, *p* < 0.001) of the variance. Introducing ToM to the model did not significantly increase the amount of variance explained (Δ*R*^2^ = 0.020, *F* (1,90) = 3.505, *p* = 0.064). Solitary behaviour (β = −0.186, *p* = 0.032) and aggressive behaviour (β = −0.543, *p* < 0.001) were significant predictors of prosocial behaviour. 

##### Victimisation

Based on the finding that victimisation was partially correlated with ToM, it was also decided to run a hierarchical multiple regression analysis with victimisation as the outcome variable (see Table 6). As shown earlier, partial correlations between ToM and victimisation were not significant for boys and girls separately, and therefore hierarchical regression was run for the whole sample. The results are shown in Table 6. The variables in the first step accounted for 67.5% of the variance (*R*^2^ = 0.675, *F* (5187) = 77.630, *p* < 0.001). This only increased to 67.6% when ToM was introduced. There was no significant change in this model (Δ*R*^2^ = 0.002, (1186) Δ*F* = 0.881, *p* = 0.349). Aggressive behaviour (β = −0.732, *p* < 0.001) and solitary behaviour (β = 0.206, *p* < 0.001) were significant predictors of victimisation. For consistency with the models of the other behaviours, interactions were not included.

##### Aggressive Behaviour

For exploratory purposes, a model was also run with aggressive behaviour as the outcome variable, but this also showed that ToM played no part in predicting aggressive behaviour. 

## 4. Discussion

The findings from the current study indicate that the different behaviours, as rated by the teaching staff, were differentially related to children’s concurrent ToM performance and that, in some cases, this relationship differed by the child’s gender. 

### 4.1. Theory of Mind and Behaviour Ratings

#### 4.1.1. Aggressive Behaviour

There was no significant relationship between aggressive behaviour and ToM score in the current study, and this pattern of results was found across boys and girls. This finding does not align with previous work, such as that by Olson et al. [28]. This could be explained by the development of aggressive behaviour. Renouf et al. [64] found that ToM relates more with indirect than aggression, and children of the ages in the current study tend to be more direct in their aggression [87]. That said, Renouf et al. [64] also suggested that there is not a direct relationship between aggression and ToM and that this may be more complex, which may account for the lack of findings in the current study.

#### 4.1.2. Victimisation

When using correlations, victimisation was negatively related to ToM. It is possible that being victimised may reduce the richness of the social environment, thus impacting the development of their ToM, or that having poorer ToM may make children more vulnerable to victimisation by their peers. In addition, it is possible that being victimised makes children less motivated to understand others’ perspectives. This finding does differ from those of Monks et al. [30], who found that there was no association between ToM and victimisation among four- to six-year-olds, whereas studies with older victims of bullying [29] have suggested that victimisation is associated with having poorer ToM. As such, this age difference may explain the difference between the current findings and those of Monks et al. [30]. In addition, Monks et al. [30] found that peer-victimisation was not a stable experience for most children during early childhood and that most children’s experiences of victimisation by peers were short-lived. Thus, they argued that children who are victimised at this age would not necessarily demonstrate some of the characteristics of those who are stably victimised in later childhood and adolescence. The difference in findings between the current study and that of Monks et al. [30] may relate to the slightly older sample in this study. It is also possible that the methodology employed may have played a role, as Monks et al. [30] employed peer-reports compared with the teacher-reports used in the current study. Although peer- and teacher-reports show some agreement [80], they do contribute unique variance [83]. It is possible that teachers may be less sensitive to the fast-paced changes in the peer-group and may therefore be more likely to report on those children for whom victimisation is a more stable experience. 

When considered alongside other behaviour variables, victimisation was not found to predict ToM, nor was ToM found to predict victimisation. This aligns more with the findings of Monks et al. [30]. Therefore, it seems that other behaviours are related to victimisation, removing this predictive relationship. It was expected that there would be some interaction between aggression and victimisation in predicting ToM. The meta-analysis by Cook et al. [63] indicated that bully-victims (children who bully others and are bullied by others) displayed lower levels of social competence than other children. However, this is not evident in the current findings. This may be because Cook et al. [63] examined bullying behaviour (rather than aggressive behaviour more generally) and did not directly assess ToM. Renouf et al. [64] reported that poorer ToM was only related to reactive, not proactive, aggression among children who had experienced victimisation. In the current study, the function of the aggression was not examined, which may account for more complex findings. It may be interesting to explore this further, using different functions of aggressive behaviour to moderate the relationship between ToM and victimisation. Therefore, it appears that victimisation and ToM are related, but there is a scope to further explore the nature of this relationship with other behaviours and across different informants. 

#### 4.1.3. Prosocial Behaviour

Prosocial behaviour was significantly and positively correlated with ToM, which is consistent with much of the previous literature [38,82,88] suggesting that prosocial behaviour is related to understanding others’ perspectives. This also aligns with the ideas of Theory Theory—that the display of these prosocial behaviours leads to a richer environment for children, thus altering and improving their ToM. However, although there was a significant positive relationship between prosocial behaviour and ToM for girls, this was not the case for boys. This is in contrast with Yagmurlu [39], who reported a significant relationship between ToM and prosocial behaviour only for boys and not girls. However, it supports and extends the findings of Walker [40] to a slightly older sample. Caputi et al. [82] suggested that children with better ToM have a superior understanding of others and are able to use this understanding to behave in a socially sensitive manner, which may include prosocial behaviour. The finding that this relationship was not present for boys is interesting. Boys tended to be rated as less prosocial than girls by teaching staff, which is in line with previous research [89], but no gender differences in ToM understanding were present in the current study. It is possible that, for boys, having a good understanding of others is not sufficient for the display of prosocial behaviour. It is possible that other factors, such as social norms for behaviour, may inhibit their display of this behaviour, even when they have similar levels of social understanding to girls. 

When prosocial behaviour was considered alongside the other types of behaviour, it did not play a significant predictive role in ToM for either gender, nor was it predicted by ToM. Therefore, whilst prosocial behaviour may be important for girls, it seems that this influence is reduced when considered alongside other behaviours. It is possible that this was influenced by the sample size in the current study, and therefore, there is a scope to explore this further with a larger group. In addition, Imuta [42] found a stronger association with prosocial behaviour and ToM as children became older, which may explain some of the contradictions in the current findings. It may be that prosocial behaviours do play a role, but when considered alongside other behaviours, this is not strong enough to reach significance. Similarly, given the importance of reciprocity in the development of a child’s environment, future research may want to consider whether being the recipient of prosocial behaviour influences ToM.

The findings derived from considering how the combined model of behaviours can predict ToM also do not tie in with the findings of Renouf et al. [62], who reported that the relationship between ToM and aggression was moderated by prosocial behaviour. Hawley [56,90,91] identified a group of individuals who employ both coercive and affiliative strategies in situations of resource control: bistrategic controllers. These individuals have been found to be popular and relatively dominant in peer groups [91,92,93]. It would be predicted that children who employ these strategies in a skillful and strategic way, deciding when to behave aggressively or affiliatively and towards whom, may demonstrate superior social understanding. However, prosocial and aggressive behaviour were not found to be significant predictors in a behaviour model predicting ToM in the current research, nor were there any interactions. This may be because there are low numbers of these children in the sample, but it is also possible that this is related to the lack of longitudinal ratings. Roseth et al. [94] explained that coercive and prosocial resource control vary within different relationships and contexts over a school year, and therefore, it may be that the nature of this research did not show how bistrategic control can relate to ToM. 

#### 4.1.4. Solitary Behaviour

Solitary behaviour was negatively related to ToM performance, suggesting that those children who remain alone show poorer performance regarding tasks assessing ToM. Whilst other actions may influence ToM, such as whether the child observes others or is disinterested [51], these findings indicate that the act of being alone is enough to relate to ToM. This aligns with what we would expect when applying Theory Theory—being alone reduces a child’s peer interactions in the social environment, therefore impacting the revision of their ToM (and vice versa). This may relate to social motivation theory, which findings have shown to relate to social competence and ToM [15]. Future research may consider whether this is moderated by the motivation for being alone. It may also be the case that a child with poorer ToM may be less popular [16] and therefore less integrated into the peer group and more likely to display behaviours that would be considered ‘solitary’.

When the relation between solitary behaviour ratings and ToM was broken down by gender, there was a difference. The negative relationship between ToM and solitary behaviour was only significant for boys and not for girls. However, there was no significant difference in the levels of solitary behaviour displayed by boys and girls. The current findings overlap with the work by Hipson et al. [95], who showed that there were more negative associations between emotion regulation and withdrawn and shy behaviour in boys than in girls. The current findings also support the work by Walker [40], who suggested that this may be related to what is viewed as socially competent behaviour for boys and girls in terms of social norms. It may be that girls who are displaying solitary behaviour are not less socially skilled, as solitary or shy behaviour may be more likely to be viewed as socially acceptable by girls. For boys, solitary behaviour may be more strongly related to lower social competence. Other researchers [96] have suggested that girls may also develop more positive relationships with their teachers. It is possible that this protects from some of the negative effects of solitary behaviour. It may also relate to the display of prosocial behaviour, which was significantly more common in girls, and the overlap between solitary and prosocial behaviour warrants further investigation with a larger sample. 

When solitary behaviour was considered as a predictor alongside other behaviours, it was found to be the only significant predictor of ToM for boys. ToM was also found to predict solitary behaviour (also only in boys). It was tentatively predicted that those who were higher in terms of solitary behaviours and aggression may have a less developed ToM. This hypothesis was based on the research indicating that children who are aggressive and withdrawn have poorer social skills [68] and more relationship problems [69,97]. However, as explained above, aggressive behaviour was not a significant predictor of ToM and was therefore not found to be related to solitary behaviour. It is possible that this relates to the collapse of both aggressive and solitary behaviours. Future research may try to examine the differential relationships between the types of solitary behaviour and aggressive behaviour, as it appears likely that these may differ [98]. 

These findings further highlight the importance of the bi-directional relationship between solitary behaviour and ToM in boys and demonstrate that this relationship is key, potentially over and above the role of other behaviours.

### 4.2. Strengths, Limitations, and Future Directions

This research considered ToM when combining behaviours and showed the importance of solitary behaviour in boys, over and above the roles of aggressive, victimised, and prosocial behaviour. When looking at behaviour types separately, it also showed relationships between prosocial behaviour and ToM (in girls only) and victimisation and ToM. These are important findings, which provide useful insight into how a wide spectrum of behaviours play a role in ToM. A unique aspect of this research was the consideration of four types of behaviour, together with the understanding of ToM, across both boys and girls. Whilst the sample size may have meant that it was not possible to detect specific interactions, using all four behaviour types within the models highlighted the importance of including a range of behaviours in the exploration of ToM and showed that solitary behaviour plays a particularly key role in boys. 

However, the current findings should be treated with caution. For instance, whilst there is a clear argument for collapsing the different subtypes of behaviours, there may be differences within these, which in turn may impact the results. Furthermore, the data in the current study were collected at one time point. Previous research has suggested that ToM may be more relevant to later behaviour [82], and longitudinal research examining the complex interrelationships between different forms of behaviour is needed to examine the relationship between children’s developing social understanding and their behaviour. In addition, whilst the sample size was adequate to run analysis on the whole sample, it is possible that low numbers of children fell into specific profiles such as bistrategic controllers or bully-victims, and a larger sample size is needed to explore these in more depth. The sample size in the current study also prevented the analysis of interactions when the sample was split by gender, meaning the effect of specific behaviour profiles may not have been detected. Given the correlations found between the various behaviour types, and considering the reciprocal nature of behaviours, it seems that the relationships between these and ToM are more complex and warrant further investigation.

It is also worth noting that traditional measures of ToM were used within the current study. Some researchers [99] have suggested that false belief ToM is related to moral judgements, which then work together to influence social judgements. As such, this approach to understanding ToM may be useful in future research into ToM and children’s behaviour.

That said, there are many strengths to this research. First, the consideration across behaviours has enabled us to understand the contribution of a range of behaviours in children’s cognitive development. Second, this study included reports from both teachers and Teaching Assistants. Teaching Assistants play an important role in the classroom [100] and the inclusion of their behaviour reports enhances the reliability of reports. Given that researchers [101,102,103] have shown that there are differences across informants’ ratings of behaviour, future research could involve using self- or peer-reports of behaviour to understand the relationship between behaviour profiles and ToM. There may also be a scope to compare reports from Teachers and Teaching Assistants. 

The findings from this study have some implications. Whilst the findings for each type of behaviour pave the way for future research, it is the findings related to solitary behaviour that may have practical implications. Much of the work that takes place in schools focuses on reducing aggressive behaviour and encouraging prosociality and social play amongst peers. However, the current findings indicate that solitary behaviour in boys plays a key role in ToM development (and vice versa). Therefore, it would be helpful to encourage more social interactions for boys, particularly in school settings, to support their cognitive development.

## 5. Conclusions

This research indicates that social cognition, as measured by ToM, is differentially related to children’s concurrent social behaviour during the formative years at school. In addition, there were some gender differences, with a less developed ToM relating to solitary behaviour for boys and not girls. Solitary behaviour in boys played an important role, over and above other behaviours, and the relationship was found to be bi-directional. Other findings highlighted the importance of prosocial behaviour in girls and some relationship between victimisation and ToM in children, but these require further exploration with a larger sample size over a longer time period. Future research into ToM could include other informants, more exploration of specific behaviour profiles, and consideration of the different forms and functions of behaviours.

## Figures and Tables

**Table 1:** Partial correlations between Theory of Mind (ToM) and behaviour ratings (controlling for age and BPVS score) by gender.

	1	2	3	4	5
1. ToM	-	0.269 *	−0.153	−0.140	−0.188
2. Prosocial	0.071	-	0.616 ***	−0.510 ***	−0.378 ***
3. Aggression	−0.041	0.352 **	-	−0.139	−0.302 *
4. Victimisation	−0.186	−0.460 ***	−0.788 ***	-	0.454 ***
5. Solitary	−0.317 **	−0.096	−0.039	0.238 *	-

Note. Female (93 df) above the diagonal, Male (92 df) below the diagonal. * *p* < 0.05, ** *p* < 0.01, *** *p* < 0.001.

**Table 2:** Hierarchical regression analysis for ToM and behaviour types.

Predictor	All Children		
	R^2^	F	β
**Block 1**	0.411	66.407 ***	
Age			0.561 ***
BPVS			0.309 ***
**Block 2**	0.458	26.204 ***	
Age			0.560 ***
BPVS			0.265 ***
Solitary behaviour			−0.145 *
Aggressive behaviour			0.052
Prosocial behaviour			0.082
Victimisation			−0.089
**Block 3**	0.476	13.612 ***	
Age			0.567 ***
BPVS			0.255 ***
Solitary behaviour			−0.123
Aggressive behaviour			0.116
Prosococial behaviour			0.070
Victimisation			−0.145
Solitary × aggressive			0.105
Solitary × prosocial			0.057
Solitary × victimisation			−0.008
Aggressive × prosocial			−0.002
Aggressive × victimisation			−0.088
Prosocial × victimisation			−0.120

Note. * *p* < 0.05, *** *p* < 0.001. Bold text used to show a new block in the hierarchical regression.

**Table 3:** Hierarchical regression analysis for ToM and behaviour types by gender.

Predictor	Boys			Girls		
	*R* ^2^	*F*	β	*R* ^2^	*F*	β
**Block 1**	0.417	33.245 ***		0.401	31.526 ***	
Age			0.544 ***			0.587 ***
BPVS			0.311 ***			0.286 ***
**Block 2**	0.490	14.239 ***		0.450	12.292 ***	
Age			0.560 ***			0.619 ***
BPVS			0.65 ***			0.234 **
Solitary behaviour			−0.145 *			−0.086
Aggressive behaviour			0.052			0.010
Prosocial behaviour			0.082			0.202
Victimisation			−0.089			0.022

Note. * *p* < 0.05, ** *p <* 0.01, *** *p* < 0.001. Bold text used to show a new block in the hierarchical regression.

**Table 4:** Hierarchical regression analysis for solitary behaviour and ToM by gender.

Predictor	Boys			Girls		
	*R* ^2^	*F*	β	*R* ^2^	*F*	β
**Block 1**	0.277	6.907 ***		0.264	6.543 ***	
Age			−0.070			−0.011
BPVS			−0.094			0.069
Aggressive behaviour			−0.590 ***			−0.285
Prosocial behaviour			−0.260 *			−0.296 *
Victimisation			0.575 ***			0.534 ***
**Block 2**	0.324	6.907 ***		0.272	5.592 ***	
Age			0.078			0.059
BPVS			−0.005			0.095
Aggressive behaviour			−0.526 **			−0.281
Prosocial behaviour			−0.265 *			−0.270 *
Victimisation			0.482 **			0.531 ***
Theory of Mind			−0.293 *			−0.113

Note. * *p* < 0.05, ** *p* < 0.01, *** *p <* 0.001. Bold text used to show a new block in the hierarchical regression.

**Table 5:** Hierarchical regression analysis for prosocial behaviour and ToM by gender.

Predictor	Boys			Girls		
	*R* ^2^	*F*	β	*R* ^2^	*F*	β
**Block** 1	0.339	9.213 ***		0.478	16.648	
Age			−0.248 **			−0.563 ***
BPVS			0.181 *			0.173 *
Aggressive behaviour			−0.114			−0.563 ***
Solitary behaviour			−0.238 *			−0.210 *
Victimisation			−0.293			0.036
**Block 2**	0.343	7.758 ***		0.497	5.592 ***	
Age			−0.198			−0.189
BPVS			0.207 *			0.123
Aggressive behaviour			−0.104			−0.543 ***
Solitary behaviour			−0.258 *			−0.186 *
Victimisation			−0.309			0.031
Theory of Mind			−0.097			−0.185

Note. * p < 0.05, ** *p* < 0.01, *** *p* < 0.001. Bold text used to show a new block in the hierarchical regression.

**Table 6:** Hierarchical regression analysis for victimisation and ToM.

Predictor	All Children		
	*R* ^2^	*F*	β
**Block 1**	0.675	77.630 ***	
Age			−0.127 *
BPVS			0.014
Aggressive behaviour			0.733 ***
Solitary behaviour			0.215 ***
Prosocial behaviour			−0.080
**Block 2**	0.676	64.797 ***	
Age			−0.097
BPVS			0.000
Aggressive behaviour			0.732 ***
Solitary behaviour			0.206 ***
Prosocial behaviour			−0.075
Theory of Mind			−0.053

Note. * *p* < 0.05, *** *p* < 0.001. Bold text used to show a new block in the hierarchical regression.

## Data Availability

The data presented in this study are available on request from the corresponding author. The data are not publicly available to ensure confidentiality.
